# 

**Published:** 1875-05-15

**Authors:** 


					﻿No. X.
MAY 16, 1876.
Vol. XVI.
THtE
Medical Izamiks:
A
.mmi-fflonlhln journal of ffldical fco'ntei.
EDITED BY
N. S. DAVIS, M. D., and F. H. DAVIS, M. D
Subscription Price, Three Dollars per Annum, in advance.
Single Numbers, Fifteen Cents.
CHICAGO.
PUBLISHED AT 65 E. RANDOLPH STREET.
				

